# Physicochemical Characteristics and Immunomodulatory Activities of Three Polysaccharide-Protein Complexes of Longan Pulp

**DOI:** 10.3390/molecules16076148

**Published:** 2011-07-21

**Authors:** Yang Yi, Sen Tai Liao, Ming Wei Zhang, John Shi, Rui Fen Zhang, Yuan Yuan Deng, Zhen Cheng Wei

**Affiliations:** 1 Ministry of Agriculture/Bio-tech Research Institute, Guangdong Academy of Agricultural Sciences, Guangzhou 510610, China; 2 College of Food Science and Technology, Huazhong Agricultural University, Wuhan 430070, China; 3 Guleph Food Research Center, Agriculture and Agri-Food Canada, 93 Stone Road West, Guelph, ON N1G5C9, Canada

**Keywords:** longan, pulp, polysaccharide-protein complex, physicochemical characteristic, immunomodulatory activity

## Abstract

Three polysaccharide-protein complexes of longan pulp (LP1-3) were isolated in this work. Their physicochemical characteristics and immunomodulatory effects on splenocytes, natural killer (NK) cells and macrophages *in vitro* were investigated. The carbohydrate portions of LP1-3 were principally composed of glucose, arabinose and mannose. LP3 displayed the maximal moisture absorption, and the thermal stability of LP2 was obviously higher than that of LP1 and LP3. All of them showed the characteristic polysaccharide and protein bands in the Fourier Transform Infrared (FTIR) spectrum. For a certain dose, all the fractions could significantly stimulate splenocyte proliferation, macrophage phagocytosis against neutral red, and NK cell cytotoxicity against YAC-1 lymphoma cell (*P* < 0.05). The results demonstrated that the polysaccharide-protein complexes of longan pulp have medical potential as immunotherapeutic adjuvants due to their immunomodulatory activities.

## 1. Introduction

The enhancement or potentiation of the host immune system has been recognized as a possible way to inhibit tumor growth without harming the host, and it is also an effective long-term healthcare strategy. Polysaccharide-protein complexes of fungi, lichens and plants have attracted extensive attention recently in the biochemical and medical fields due to their immunological activities and relatively low toxicities [1]. As important bioactive compounds of *Ganoderma lucidum*, polysaccharide-protein complexes exhibit immunomodulatory and antiherpetic activities, and their mass ratios of carbohydrate/protein range from 10.4 to 28.9 [2,3,4]. The complexes of *Lycium barbarum* can upregulate cytokine expression in human peripheral blood mononuclear cells and improve the immune system of S180-bearing mice [1,5,6], and that of *Lentinus edodes* possesses remarkable antifatigue effects [7]. Moreover, the polysaccharide-protein complex of *Agaricus blazei Murill* can stimulate the non-specific complements and humoral immune functions via the action of polyclonal activators of B cells, inhibition of tumor growth and metastasis, and up-regulation of dendritic cell maturation [8]. However, the biological activity of polysaccharide-protein complex from longan (*Dimocarpus longan* Lour.) fruit is still not available.

Longan is a commercially attractive fruit which is widely distributed in subtropical areas. Longan pulp has been used as a traditional Chinese medicine to promote blood metabolism, soothe nerves, relieve insomnia, and prevent amnesia [9,10]. Several studies have been conducted on the bioactivity of longan extract, such as memory-enhancing [9], anxiolytic [11], antioxidant and immunomodulatory activities [12]. Many reports have focused on the polysaccharides from longan fruit pericarp, which were related to the functions of antioxidant [13,14], anticancer [13], antityrosinase [15] and anti-glycation [16]. Zhong *et al.* [17] have investigated the bioactivity of longan pulp polysaccharides using S180 tumor mice models. The results demonstrated that the polysaccharides possessed effective free radical scavenging, immunity-modulation and antitumor actions. However, information on polysaccharide-protein complex from longan pulp is limited. Although the pulp is not the part of longan fruit with the highest polysaccharide content, it is the only edible part which is widely used in traditional medicine. In order to find out more about the theoretical basis of the health-promoting effect of longan, the polysaccharide-protein complexes were first isolated from the longan pulp aqueous extract (*i.e.*, longan polysaccharide-protein complex, LP), and their physicochemical characteristics and immunomodulatory effects on murine immunocytes *in vitro* were then investigated. 

## 2. Results and Discussion

### 2.1. Physicochemical characteristics of LP1-3

#### 2.1.1. Characterization of polysaccharide-protein complex

**Figure 1:**
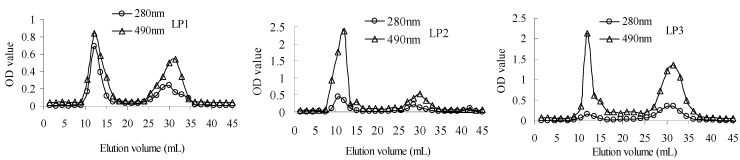
Gel column chromatograms of LP1-3. Polysaccharides and proteins were detected by phenol-sulfuric acid method at 490 nm and UV measurement at 280 nm, respectively.

Gel column chromatograms of polysaccharides and proteins in LP1-3 are shown in Figure 1, which corrresponds to the curves recorded at 490 and 280 nm, respectively. In the chromatograms, the responses of polysaccharides and proteins match, and both of them have two main peaks indicating two components in each sample. The results manifested that three fractions isolated from longan pulp displayed polysaccharide-protein complex characteristics. Moreover, the peak area ratios of polysaccharides to proteins of LP1-3 were respectively 1.5, 4.1 and 4.7, indicating the highest protein content of LP1.

**Figure 2:**
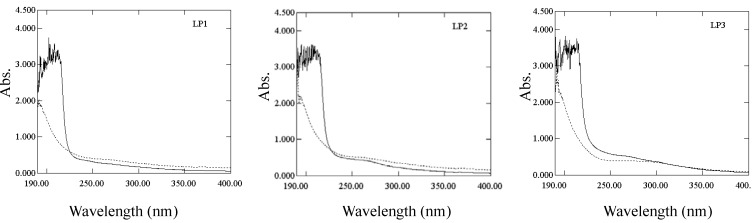
UV spectra of β-elimination reactions of LP1-3. “–” represents the absorption of LP treated with sodium hydroxide, and “-” represents the absorption of non-treated LP.

The *β*-elimination reaction of polysaccharide-protein complex was used for the detection of O-glycosidic bonds. The O-glycosidic bond between serine (or threonine) and sugars, produced α-aminoacrylic acid and α-aminobutenoic acid under the treatment of weak base, which resulted in the increase of absorbance at 240 nm [18]. Only the absorption of LP3 treated with sodium hydroxide was higher than that of non-treated control at 240 nm (as shown in Figure 2), which indicated the presence of O-glycosidic bonds.

#### 2.1.2. Chemical composition

Chemical compositions of LP1-3 are summarized in Table 1. The contents of neutral polysaccharides, hexuronic acids and proteins in LP1-3 were significantly different between the samples (*P *< 0.05), and their ratios of carbohydrates to proteins were 1.91:1.00, 15.50:1.00 and 29.61:1.00, respectively. Moreover, the monosaccharide composition of LP1 was obviously different from LP2 and LP3. LP1 was mainly composed of glucose and arabinose, but LP2 and LP3 were mainly composed of glucose and mannose. LP3 has the highest content of glucose. Xylose was observed to be an important monosaccharide in LP2. Sixteen amino acids were measured in the complexes, and the hydrophobic amino acid/protein ratios of LP1, LP2 and LP3 were 1.00:2.26, 1.00:2.44 and 1.00:2.81, respectively. Besides the presence of binding proteins of LP1-3, their monosaccharide compositions are all different from the pulp (cv. Fenglisui) polysaccharides isolated by Yang *et al.* [10] and the pericarp (cv. Shixi) polysaccharides purified by Yang *et al*. [19]. Different material and/or separation method used could account for the differences in composition.

**Table 1:** Chemical compositions of LP1-3 (g/100g).

Composition	LP1	LP2	LP3	Composition	LP1	LP2	LP3
Neutral polysaccharide	62.35 ± 2.12 ^a^	82.45 ± 2.42 ^b^	90.44 ± 2.12 ^c^	Protein	33.72 ± 0.16 ^c^	5.91 ± 0.07 ^b^	3.20 ± 0.06 ^a^
Hexuronic acid	1.98 ± 0.14 ^a^	9.03 ± 0.40 ^c^	4.31 ± 0.20 ^b^	Aspartate	3.90	0.73	0.31
Percentage (%)				Glutamic	4.34	0.96	1.00
Ribose	3.29	0.81	1.32	Serine	1.74	0.25	0.12
Rhamnose	2.91	0.40	0.12	Glycine	1.78	0.36	0.16
Arabinose	28.2	6.63	3.76	Threonine	1.86	0.29	0.12
Xylose	0.55	9.44	0.51	Histidine	0.86	0.10	0.05
Mannose	7.20	27.3	28.9	Alanine *	2.58	0.62	0.41
Glucose	46.8	54.6	63.9	Arginine	1.62	0.31	0.09
Galactose	11.0	0.87	1.50	Tyrosine *	1.22	0.09	0.02
Molar ratio				Valine *	2.49	0.41	0.16
Ribose	0.55	0.12	0.35	Metione	0.75	0.05	<0.05
Rhamnose	0.44	0.06	0.03	Phenylalanine *	1.90	0.24	0.07
Arabinose	4.70	1.00	1.00	Isoleucine *	1.89	0.29	0.09
Xylose	0.09	1.42	0.14	Leucine *	3.34	0.49	0.20
Mannose	1.00	3.43	6.40	Lysine	1.98	0.46	0.16
Glucose	6.50	6.86	14.16	Proline *	1.48	0.28	0.19
Galactose	1.83	0.13	0.40				

The contents of neutral polysaccharide and hexuronic acid were individually determined by the phenol-sulphuric acid method and the method of Blumenkrantz and Asboe-Hansen. The monosaccharide and amino acid compositions of LP1-3 were determined by gas chromatograph/mass spectrometer and reverse phase-high performance liquid chromatography, respectively. The values for neutral polysaccharide, hexuronic acid, and protein were expressed as mean ± SD (n = 3). The results having the different letter in each row are significantly different (*P* < 0.05), * represents hydrophobic amino acid.

#### 2.1.3. Moisture absorption and thermodynamic property

**Figure 3:**
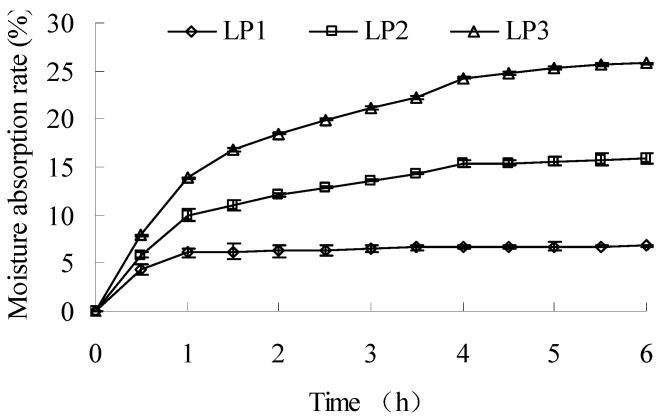
Moisture absorption curves of LP1-3.

The moisture absorption curves of LP1-3 are shown in Figure 3. The absorption rate of LP1 at 6 h was significantly lower than that of LP2 (*P *< 0.01), and LP3 showed the highest absorption rate (*P *< 0.01). The time needed for constant moisture levels of LP1-3 were stabilized for 1, 4 and 5.5 h, respectively. 

**Figure 4:**
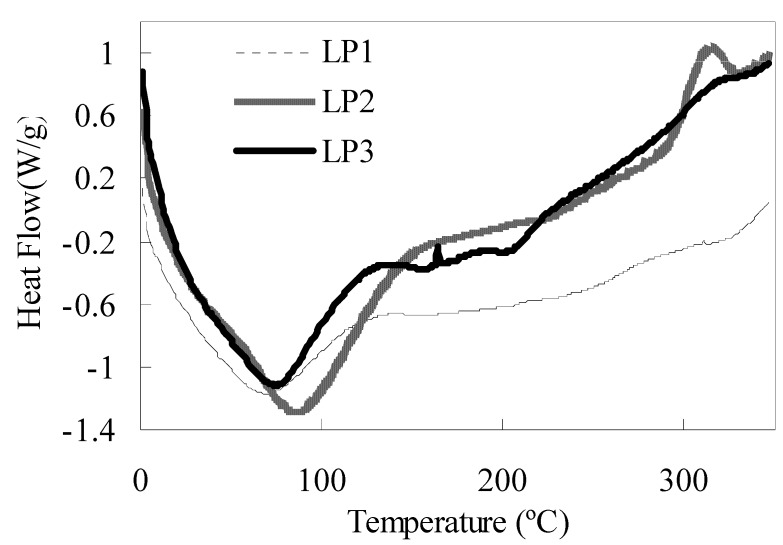
DSC curves of LP1-3.

The DSC curves in Figure 4 show the thermodynamic properties of LP1-3. Each curve had a heat absorption peak in the temperature range of 10–150 °C, and the peak temperatures of LP1-3 were 64.96, 82.26, and 69.07 °C, respectively. LP2 had a heat emission peak at 314.71 °C, and LP3 has a weak heat emission peak at 165.93 °C. The heat absorption corresponded to the liberation of adsorbed water, and the heat emission corresponded to the pyrolysis of polysaccharide-protein complex [20]. 

The separation of LP1 might be related to the effect of isoelectric point of binding proteins in the dialysis procedure. Its low-polysaccharide/high-protein composition could contribute to its lower absorption rate and thermostability. LP2 was prepared by ethanol-induced precipitation, and this procedure might cause the formation of inter-/intra-molecular hydrogen bonds and the decrease of the exposed hydrophilic group for polysaccharide molecules. The resulting change of molecular microstructure, which was reflected by the different physical properties, accounts for the weaker hydrophilicity and better thermostability of LP2 compared with that of LP3. Moreover, the highest polysaccharide content also importantly contributed to the highest absorption rate of LP3.

#### 2.1.4. FTIR spectrum characterization

The FTIR spectra of LP1-3 were determined using a Fourier transform infrared spectrophotometer in the frequency range of 4000–400 cm^−1^. The absorptions, functional groups and structural characteristics of the FTIR spectra of LP1-3 are summarized in Table 2. All of the complexes showed the characteristic bands of polysaccharides, including the hydroxyl group bands in the 3600–3200 and 1075–1010 cm^−1^ range, the alkyl group bands at about 2926, 2850 and 1458 cm^−1^, and the carboxyl group bands in the 1740–1210 cm^−1^ region (characteristic of uronic acid). Meanwhile, the amino group band at 3460–3150 cm^−1^ and 1650–1500 cm^−1^ implied the presence of proteins [21]. However, Table 2 exhibited some differences among the spectra of three complexes, and the structural information of LP1 was lacking because of the lack of absorption between 960–740 cm^−1^. Notably, some structural information of FTIR spectrum was uniform. Li *et al.* [22] indicated that the absorption peaks at about 920 and 850 cm^−1^ might be characteristic of (1→4)-α-glucans. Ruan *et al.* [23] deemed the band at 920 cm^−1^ to show C–O–C stretching of β-glucopyranoses. Moreover, Shi *et al.* [24] regarded that α-pyranoses had the band absorption at about 850 cm^−1^. The absorptions at about 1050 cm^−1^ indicated the α-pyranose form of the glucosyl residue or the bending vibration of hydroxyl groups [22,25]. The absorptions at about 770 cm^−1^ suggest the symmetrical ring vibration of D-glucopyranose rings, which might be due to the α-configuration of rhamnose units [26]. 

**Table 2:** FTIR spectrum analysis of functional groups of LP1-3.

Absorption (cm^−1^)			Functional group	Structural characteristic	
LP1	LP2	LP3			
3398.0	3431.8	3430.9	hydroxyl group (-OH)		stretching vibration of O-H
			amino group (-NH_2_)		stretching vibration of N-H
2924.6,2853.1	2928.0	2930.4	alkyl group (-CH_2_-)		stretching vibration of C-H
1742.9			carboxyl group (-COOH), aldehyde group (-CHO) or esterfunction (-COOR)		stretching vibration of C=O
1654.5	1638.0	1647.4	carbonyl group (-C=O or -CHO)		stretching vibration of C=O
			amide group (-NH_2_ or –COR)		bending vibration of N-H or stretching vibration of C=O
			amino group (-NH_2_)		bending vibration of N-H
			bound water		
1541.3			amino group (-NH_2_) or amide group (-NH_2_)		bending vibration of N-H
			carbonyl group (-C=O)		stretching vibration of C=O
1457.9	1458.8	1458.0	alkyl group (-CH_2_- or –CH_3_)		bending vibration of C-H
1418.8	1425.2		carboxyl group (-COOH)		stretching vibration of C-O
1378.2	1364.2	1363.6	carboxyl group (-COOH)		symmetrical stretching vibration of C=O
1237.7	1275.1, 1209.1	1270.0	carboxyl group (-COOH)		bending vibration of O-H
1159.1	1157.9		ether (-C-O-C-)		stretching vibration of C-O
1053.2	1043.9	1013.1	hydroxyl group (-OH)		bending vibration of O-H
	918.0	920.8	D-glucopyranose ring		antisymmetrical ring vibration
		865.0	furanose		
	847.9		α-type glycosidic linkage		bending vibration of C-H
		819.6	α-D-galactopyranose		
	764.2	776.2	D-glucopyranose ring		symmetrical ring vibration

The absorption at about 1540 cm^−1^ corresponds to the secondary–CONH–group of proteins in LP1 [27], and absorptions at about 815 cm^−1^ might be due to the presence of α-type glycosidic linkages [28]. Therefore, LP2 and LP3 were mainly composed of glucosyl residues in the α-pyranose form. The results were consistent with the conclusion of Yang *et al. *[10], *i.e.*, longan polysaccharides were β-type acidic heteropolysaccharides with pyran groups. Information about functional groups and structural characteristics were gleaned from the book of Zhang [21].

### 2.2. Immunomodulatory activities of LP1-3

#### 2.2.1. Effects on splenic lymphocyte proliferation

**Figure 5:**
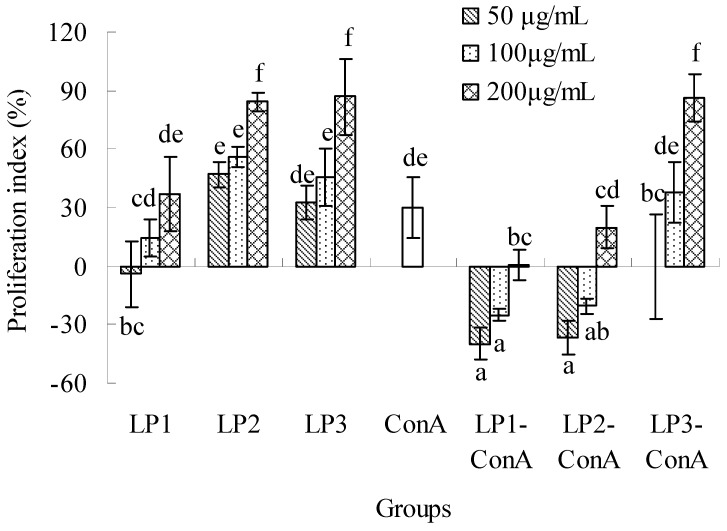
Effects of LP1-3 on the proliferation of splenic lymphocytes. The proliferation was assessed by MTT assay and expressed as means ± SD (n = 3). The statistically significant differences among the groups were evaluated with ANOVA followed by the S-N-K test. Different letters in the figure represent the statistical difference at *P* < 0.05.

The effects of LP1-3 on splenic lymphocyte proliferation are shown in Figure 5. LP1-3 could obviously stimulate the proliferation in the dose range of 50–200 μg/mL (except 50 μg/mL of LP1), and the proliferation index ranged from 14.6–86.8%. Their effects were in a dose-dependent manner. When the same dose was used, the proliferations of LP2 and LP3 were almost the same (*P* > 0.05), but significantly higher than that of LP1 (*P *< 0.05). ConA could stimulate the proliferation of lymphocytes with a 30.0% increase. However, the coexistence of LP and ConA significantly decreased the lymphocyte proliferation (except 100 and 200 μg/mL of LP3) compared with the ConA group or the same dose of LP group without ConA (*P *< 0.05). The results were similar to the report of polysaccharides from five *Ganoderma *species by Chen *et al*. [29]. Interestingly, the previous studies have confirmed that polysaccharides or polysaccharide-protein complexes from *Ganoderma lucidum* selectively stimulate B cells, but not T cells [3,30,31]. Our previous tests had found that the lymphocyte proliferation was significantly inhibited by the coexistence of 5 μg/mL ConA and 5 μg/mL lipopolysaccharide (data not shown). It was suggested that LP1-3 had a strong induction effect on B cell proliferation due to the lipopolysaccharide action, but the potential mechanisms involved in the inhibition of ConA-induced proliferation and the difference of activity were not clear for LP1-3. 

#### 2.2.2. Effects on NK cell cytotoxicity

**Figure 6:**
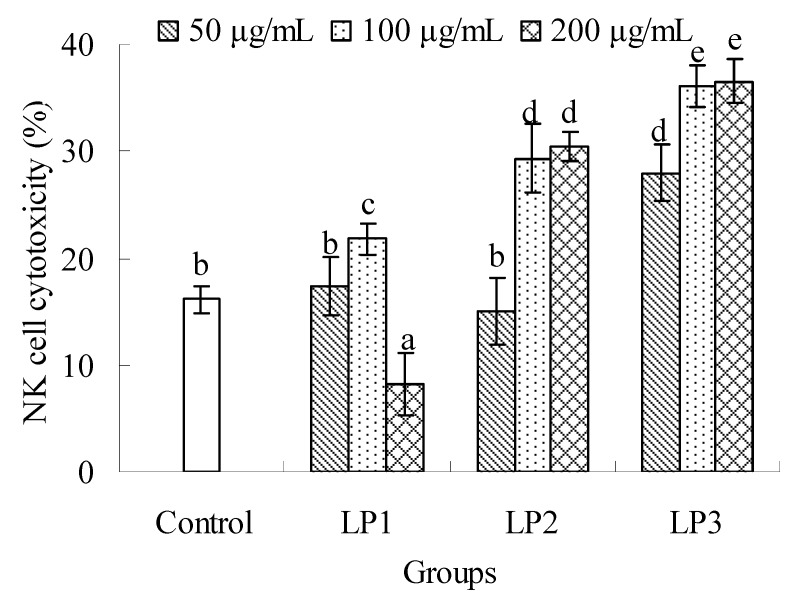
Effects of LP1-3 on the cytotoxicity of NK cells. NK cell cytotoxicity was assessed by MTT assay and expressed as means ± SD (n = 3). The statistically significant differences among the groups were evaluated with ANOVA followed by the S-N-K test. Different letters in the figure represent the statistical difference at *P* < 0.05.

As seen in Figure 6, all the complexes could enhance the NK cell cytotoxicity against YAC-1 cell at an adaptive dose compared with the control. The NK cell cytotoxicity of LP1 was significantly reduced at 200 μg/mL (*P* < 0.05). LP2 could enhance the activity at 100 and 200 μg/mL (*P* < 0.05), and no significant difference was found between the two doses (*P* > 0.05). LP3 could promote the NK cell cytotoxicity in the dose range of 50–200 μg/mL (*P* < 0.05), which exhibited the best capability to improve the NK cell cytotoxicity among three samples tested. The special structures of LPs might differ from each other due to their different protein content, monosaccharide composition and molecular weight, which resulted in their different effects on the cytotoxicity of NK cells. It was found that polysaccharides usually showed bidirectional regulation on immune system at low and high dose. It was also possible that 100 μg/mL of LP1 was the highest dose where LP1 could up-regulate the cytotoxicity of NK cells. However, the highest doses where LP2 and LP3 showed up-regulation might be higher than 200 μg/mL. Therefore, cytotoxic down-regulations of LP2 and LP3 were not found.

#### 2.2.3. Effects on macrophage phagocytosis

**Figure 7:**
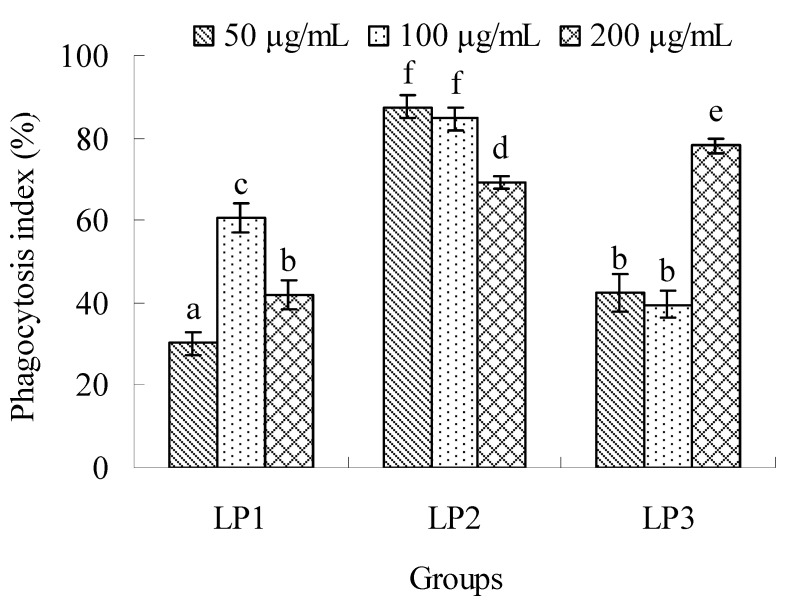
Effects of LP1-3 on the phagocytosis of macrophages. The activity was evaluated by the phagocytosis index of neutral red and expressed as means ± SD (n = 3). The statistically significant differences among the groups were evaluated with ANOVA followed by the S-N-K test. Different letters in the figure represent the statistical difference at *P* < 0.05.

The effects of LP1-3 on the phagocytosis of macrophages are shown in Figure 7. All the complexes could enhance macrophage phagocytosis in the dose range of 50–200 μg/mL, and their phagocytosis indexes were in the range from 30.15% to 87.60%. The optimal doses of LP1-3 were found at 100, 50 and 200 μg/mL, respectively. The maximal phagocytosis ratios were exhibited at 100 and 50 μg/mL of LP2, and no significant difference was found between them (*P *> 0.05). The polysaccharide-protein complexes of *Dipsacus asperoides *inhibited the phagocytosis of macrophages, and the inhibitor was proved to be the binding proteins [32]. The binding proteins of LP1 might unimportantly or negatively contribute to the phagocytosis. 

Five polysaccharide-protein complexes from *Lycium barbarum* L had different carbohydrate to protein ratios, whereby higher protein complexes showed better effect on T cell activation, and higher polysaccharide complexes exhibited better effect on the activation of dendritic cells and macrophages. Meanwhile, the T cell-stimulatory effects of higher protein complexes were significantly decreased when the proteins were digested [5]. LP1-3 significantly exhibited enhancement on splenocyte proliferation, NK cell cytotoxicity and macrophage phagocytosis *in vitro*, and the immunomodulatory activities of LP2 and LP3 were better than that of LP1 in the dose range of 50–200 μg/mL. The results implied that the potential active component was the polysaccharides. The stimulating activity of polysaccharide triggered by the recognization of cell receptor partly depends on monosaccharide composition. Lo *et al.* [33] deemed that bioactive polysaccharides or polysaccharide-protein complexes were mainly composed of galactose, rhamnose and arabinose with the molecular mass more than 100 kDa. However, LP1, which displayed the weakest immunomodulatory activities, contained the highest contents of galactose, rhamnose and arabinose. Lo *et al.* [34] considered that arabinose, mannose, xylose and galactose played an important role in the stimulation of macrophage, but not glucose. In addition, mannose had positive effects on the immunomodulatory activity of polysaccharide of *Aloe vera* [35]. It was implied that mannose importantly contributed to the immunomodulatory activities of LP2 and LP3. Moreover, the better activities of LP2 and LP3 might be partly related to their higher water-solubility [34]. 

## 3. Experimental

### 3.1. Plant materials

Fresh fruits of longan (cv. Chu-liang) at the mature stage were provided by Pomology Research Institute of Guangdong Academy of Agricultural Sciences (Guangzhou, China). Fruits were selected on the basis of shape and colour, and then dried in an oven at 50 °C for a week. The pulp was then stripped manually and grinded by a mill (A11 basic, ZKA-Werke, Germany). The generated powder was packaged and stored at −20 °C.

### 3.2. Preparation of LP

The extraction and purification of LP were implemented according to our previously reported method [36,37]. Dried longan pulp powder (50 g) was extracted with distilled water (1:40 ratio, w/v) containing 2000 U/g of cellulase (Fluka AG, Buchs, Switzerland) in an ultrasonic cell disintegrator (JY92-II,Xinzhi Bio-technology and Science Inc.,Lingbo, China) for 60 min under the condition of 55 °C, pH 5.0 and 250 W. The temperature was then raised to 80 °C and maintained for 2 h. After that, the extract was collected by centrifugation at 4,500 rpm for 10 min. The pigments and proteins in the supernatant were removed by the static absorption on anion exchange resin D301-F (4:25 ratio, w/v, Jiangsu Suqing Water Treatment Engineering Group Co., Ltd., Jiangyin, China) at 50 °C and pH 5 for 2 h. The treated extract was filtered through a Whatman No.1 paper and concentrated at 55 °C by using a vacuum rotary evaporator (BC-R203, Shanghai Biochemical Equipment Co., Shanghai, China). The concentrate was dialyzed against distilled water for 5 days, and then centrifuged at 4,500 rpm for 10 min. The precipitate was harvested and lyophilized as LP1. The supernatant was concentrated and separated into two equal volumes. To one of them was added three volumes of dehydrated ethanol overnight at 4 °C to precipitate polysaccharides, and the generated precipitate was lyophilized as LP2. Another one was directly lyophilized as LP3. The yields of LP1-3 were 2.14%, 6.85% and 9.77%, respectively. The samples were packaged and kept in a desiccator at room temperature. 

### 3.3. Characterization analysis

#### 3.3.1. Characteristics of polysaccharide-protein complex

*Gel column chromatography: *LP (2 mg) was dissolved in distilled water (1 mL). After centrifugation at 4,500 rpm for 15 min, the supernatant was injected onto a Sephadex G-100 gel filtration chromatography column (20 × 1.5 cm) followed by the elution with distilled water at a flow rate of 0.1 mL/min. The eluants (1.5 mL/tube) were collected. The concentrations of polysaccharides and proteins in the eluants were analyzed by the phenol-sulfuric acid method at 490 nm [38] and UV measurement at 280 nm, respectively.

*β-Elimination reaction:* LP (2 mg) was dissolved in distilled water (2 mL). After centrifugation at 4,500 rpm for 15 min, the supernatant was mixed with distilled water (2 mL) or 0.4 mol/L sodium hydroxide (2 mL) and incubated at 45 °C for 1.5 h. The UV spectrum of the mixture was then measured in the wavelength range of 190–400 nm. 

#### 3.3.2. Chemical composition analysis

*General analysis:* The content of neutral polysaccharide was determined by yhe phenol-sulphuric acid method [38] and expressed as glucose equivalents. The content of hexuronic acid was determined by the method of Blumenkrantz and Asboe-Hansen [39] and expressed as glycuronic acid equivalents. 

*Gas chromatograph-mass spectrometry (GC-MS):* A GC-MS method was applied for identification and quantification of monosaccharide in LP. Briefly, LP (40 mg) was hydrolyzed at 100 °C for 6 h in 2 M sulphuric acid (10 mL). Saturated barium hydrate was added for neutralizing the excess sulphuric acid. The hydrolysate was filtered through 0.2 μm syringe filters (Whatman, UK), dried under a stream of N_2_, and fully mixed with hydroxylamine hydrochloride (70 mg)/pyridine (5 mL) at 90 °C for 60 min. Acetic anhydride (5 mL) was added into the tube containing room-temperature reaction mixture. The acetylated hydrolysates in the mixture were extracted by trichloromethane followed by evaporation under a stream of N_2_. The final product (1 mL) was analyzed by GC-MS using a DB-1 column (15 m × 0.2 mm, 0.33 μm, J&W Scientific) and the following temperature programme: the initial temperature of column was 100 °C, then increased to 280 °C at a rate of 10 °C/min, and kept for 15 min at 280 °C; injection temperature was 280 °C. This procedure was performed on an Agilent 6890 GC coupled with 5973 MS. The temperature of mass spectrometer ion source was 230 °C. Sample (1 µL) was injected into the column with the split ratio of 10:1.

*Reverse phase-high performance liquid chromatography (RP-HPLC):* An online pre-column derivatization coupled with HPLC analysis method were employed for determination of amino acids in polysaccharide-protein complex using *ortho*-phthalaldehyde (OPA) and 9-fluorenylmethyl chloroformate (FMOC-Cl) as derivatizing agents. Briefly, LP (20 mg) was accurately weighed into a screw-capped tube and 6 M hydrochloric acid was added. The tube was sealed and hydrolyzed for 22 h at 110 °C. After hydrolysis, the sample was introduced into the online derivatization system. The analysis was performed on Agilent 1100 series apparatus within a Hypersil ODS column (dimensions 4.0 × 125 mm and particle size 5 μm) and a programmable fluorospectrophotometer detector. The binary gradient elution was linear over 25 min from 0% to a final composition of 100% mobile B. The column was maintained at 40 °C and the flow rate at 1.0 mL/min. Mobile phase A was sodium phosphate buffer (PB, 10 mmol·L^−1^, pH 7.2) containing 0.5% (φ) tetrahydrofuran, while mobile phase B was PB-methanol-acetonitrile (volume ratio, 50:35:15). OPA derivatives were detected by the programmable fluorometer with excitation (λ_ex_) and emission (λ_em_) wavelengths set at 340 and 450 nm, respectively. The FMOC derivatives were detected at λ_ex_ 260 nm and λ_em_ 305 nm, the wavelength change occurred at 20.5 min.

#### 3.3.3. Moisture absorption

LP (0.10 g) was measured in a dish and kept in a desiccator at room temperature for 3 days. The dish was then placed in an open container at room temperature, and measured at an interval of 30 min till a constant weight was reached. The moisture absorption rate (%) was expressed as the ratio of increased mass to initial mass.

#### 3.3.4. Differential scanning calorimetry (DSC)

LP (10 mg) was crimp-sealed in an aluminum case, and an empty one was used for reference. DSC curves were scanned in the temperature range of 1–350 °C at a heating rate of 10 °C/min. 

#### 3.3.5. Fourier transform infrared spectrum (FTIR)

The FTIR spectrum of the sample was determined using a FTIR spectrophotometer (Nexus 5DXC FTIR, Thermo Nicolet, USA). The sample was grounded with spectroscopic grade potassium bromide (KBr) powder and then pressed into a 1 mm pellet for FTIR measurement in the frequency range of 4000–400 cm^−1^.

### 3.4. Immunomodulatory activity analysis

#### 3.4.1. Animals and cells

Specific pathogen-free Kunming mice (male, 20.0 ± 2.0 g, certificate number: SCXK-Yue 2006-0015) were purchased from Laboratory Animal Sciences Center of Southern Medical University (Guangdong, China). The mice were bred on a 12-h-dark/12-h-light cycle at 22 ± 2 °C and allowed free access to standard laboratory rodent diet (Laboratory Animal Sciences Center of Southern Medical University, China) and tap water. 8–12 week-old mice were sacrificed by cervical dislocation, and their spleens removed aseptically and then minced in aseptic phosphate-buffered saline (PBS). The splenic cells were harvested through sterilized meshes (200 mesh) at room temperature. After the red blood cells were removed by hemolytic Gey's solution, the remaining cells were washed twice and resuspended in RPMI 1640 complete medium (Gibco BRL, Grand Island, NY, USA) containing 10% fetal bovine serum (Gibco BRL). The cell concentration was adjusted to 1 × 10^7^ cells/mL. The resident macrophages of mice were harvested by peritoneal lavage, and their cells were cultured in complete medium at the concentration of 2 × 10^6^ cells/mL. The experimental procedures were approved by the laboratory animal committees of Guangdong Province. All the animal treatment was performed in accordance to the Guide for the Care and Use of Laboratory Animals. YAC-1 lymphoma cell line was provided by Experiment Animal Center of Sun Yat-sen University (Guangzhou, China) and used as target cell at the concentration of 5 × 10^5^ cells/mL.

#### 3.4.2. Splenic lymphocyte proliferation

The assay of splenic lymphocyte proliferation was implemented according to the method described by Li *et al.* [40] and Wang *et al. *[41]. Splenocyte suspension (50 μL/well) was plated in a 96-well culture plate with or without concanacalin A (5.0 μg/mL, ConA, Sigma, St. Louis, MO, USA). The filter-sterilized samples (final concentration: 50, 100 and 200 μg/mL) were added into the cell well. After incubation for 68 h (37 °C, 5% CO_2_), each well was added in 5 mg/mL final concentration of 3-[4,5-dimethylthiazol-2-yl]-2,5-diphenyltetrazolium bromide (MTT, Sigma). The plate was further incubated for 4 h, and acidified isopropyl alcohol (100 μL/well) was then added and kept for 12 h to dissolve the formazan crystals. Finally, the plate was analyzed at 570 nm using a microplate reader (Thermo Labsytems, Helsinki, Finland). The proliferation index (%) was calculated as: (OD_S_ – OD_C_)/ OD_C_ × 100 in which OD_S_ and OD_C_ represented the OD values of stimulated well and control well, respectively. Data was expressed as mean ± SD of triplicate experiments.

#### 3.4.3. Natural killer cell cytotoxicity

Splenocytes was prepared as the effector cell for splenic NK cell activity assay as described by Wang *et al. * [41]. Splenocyte suspension (50 μL/well) was plated in a 96-well culture plate with sample (30 μL, final concentration: 50, 100 and 200 μg/mL) or complete medium for 24 h of incubation (37 °C, 5% CO_2_). Target cells (20 μL) were then added to be the experimental group. The wells with splenocytes and YAC-1 cells were the effector control and target control, respectively. The plate was incubated for 4 h, followed by another 4 h with MTT (5 mg/mL). Then, each well was added in 100 μL acidified isopropyl alcohol, and measured 12 h later at 570 nm. The NK cell cytotoxicity (%) was calculated as: [OD_T_ – (OD_exp_ – OD_E_)]/ OD_T_ × 100, where OD_exp_, OD_E_ and OD_T_ represented the OD values of experimental group, effector control and target control, respectively. Data was expressed as mean ± SD of triplicate experiments.

#### 3.4.4. Macrophage phagocytosis

Macrophage suspension (100 μL/well) was plated in a 96-well culture plate and incubated for 3 h (37 °C, 5% CO_2_). The adherent macrophages were washed twice by complete medium and then incubated with samples (final concentration: 50, 100 and 200 μg/mL) for 24 h. The stimulated cells were washed twice by PBS, and Neutral Red (100 μL, 0.1%, w/v) was then added. The plate was incubated for 4 h. After the removal of unphagocytized neutral red by PBS, cell lysate (100 μL, the volume ratio of acetic acid to ethanol was 1:1) was added and the mixture kept for 12 h. The OD value of each well was measured at 570 nm. The phagocytosis index (%) was calculated as: (OD_S_ – OD_C_)/ OD_C_ × 100 in which OD_S_ and OD_C_ represented the OD values of stimulated well and control well, respectively [41]. Data was expressed as mean ± SD of triplicate experiments.

### 3.5. Statistical analysis

The data were expressed as means ± standard deviations. Significance of difference was evaluated with one-way ANOVA, followed by the Student-Newman-Keuls test by SPSS 11.5 software. *P*-value of 0.05 was used as the threshold for the significance. 

## 4. Conclusions

Three water-soluble fractions (LP1-3) were isolated from longan pulp, which were demonstrated as polysaccharide-protein complexes with the carbohydrate portions principally composed of glucose, arabinose and mannose. In addition, LP1-3 were found to exhibit immunomodulatory effects on splenocytes, NK cells and macrophages, and their activities were different, due to their varied physicochemical characteristics. The structure-function relationship of longan polysaccharide-protein complex involved in immunomodulation is complicated. Further research on the structural features and mechanism of immunomodulatory activity of LP is suggested to be needed.
